# Acute Reactive Acalculous Cholecystitis Secondary to Duodenal Ulcer Perforation

**DOI:** 10.7759/cureus.4331

**Published:** 2019-03-27

**Authors:** Shab E Gul Rahim, Mohammad Alomari, Shrouq Khazaaleh, Ahmed Alomari, Laith A Al Momani

**Affiliations:** 1 Internal Medicine, Cleveland Clinic - Fairview Hospital, Cleveland, USA; 2 Internal Medicine, Cleveland Clinic Foundation, Cleveland, USA; 3 Internal Medicine, The Hashmite University, Al-Zarqa, JOR; 4 Internal Medicine, East Tennessee State University, Johnson City, USA

**Keywords:** acute acalculous cholecystitis, duodenal ulcer, cholelithiasis, prostaglandins

## Abstract

Acute cholecystitis is the inflammation of the gallbladder, classically caused by gall stones obstructing the cystic duct. In contrast, acalculous cholecystitis is a gallbladder inflammation occurring in the absence of cholelithiasis with a reported prevalence of 10% of all cases of acute cholecystitis. Reactive acalculous cholecystitis is an extremely rare subset of this disease that results from an adjacent inflammatory or infectious intra-abdominal process that may lead to gallbladder stasis, ischemia, and subsequent wall inflammation. Many factors have been associated with acalculous cholecystitis, including (but not limited to) hemodynamic instability, altered immunity, and biliary tree anomalies. Lack of specific signs and symptoms of this particular entity often delays the diagnosis. Herein, we present a rare case of acute, reactive, acalculous cholecystitis secondary to a perforated duodenal ulcer found incidentally during laparoscopic cholecystectomy.

## Introduction

Acute cholecystitis, as the name implies, is an inflammation of the gallbladder. It is secondary to gallstones in 90% - 95% of the cases where, classically, ductal obstruction by the gall stone leads to distension and edema of the gallbladder [1]. Only 5% - 14% of the cases of acute cholecystitis are acalculous [1] and are associated with risk factors, such as immunosuppression, ampullary stenosis, choledochal cyst, severe hypotension, sepsis, ischemia, and total parenteral nutrition (TPN) [2-7].

Herein, we report the case of a perforated duodenal ulcer-causing reactive acalculous cholecystitis. This particular presentation is rarely encountered and is typically diagnosed during laparoscopic cholecystectomy with a reported incidence of 0.13% in one study [8]. Typically, patients with a perforated peptic ulcer present with sudden, severe, and diffuse abdominal pain. However, atypical presentations have also been described in the literature [9], especially if the perforation was walled off and/or associated with extensive local fibrosis. Gallbladder changes, in this case, are mostly thought to be a reactive phenomenon secondary to the nearby inflammation caused by a perforated ulcer.

## Case presentation

A 76-year-old female with a past medical history significant for hypertension and dyslipidemia was admitted to our hospital for generalized weakness and decreased oral intake. She reported a weight loss of approximately 20 pounds over the course of 45 days. She denied any fevers, chills, and upper or lower gastrointestinal symptoms.

She was being evaluated by her primary care doctor for abnormal liver enzymes. Her prior to admission medications included a statin and vitamin C. The statin was stopped by the primary care provider in lieu of the abnormal liver function tests. She denied any use of over-the-counter herbal medications or acetaminophen.

Other than the signs of dehydration, the remainder of the physical examination was unremarkable. Laboratory workup showed a normal white blood cell count of 9.6 K/uL (normal range (NR): 3.70 - 11.00 k/uL), hemoglobin of 15.3 g/dL (NR 11.5 - 15.5 g/dL), hypokalemia of 2.7 mEq/L (NR 3.7 - 5.1 mmol/L), and mild hypochloremia of 92 mEq/L (NR 97 - 105 mmol/L). Blood urea nitrogen (BUN) was 24 mg/dL (NR 7 - 21 mg/dL) and creatinine was 0.92 mg/dL (NR 0.58 - 0.96 mg/dL). Liver enzymes were found to be elevated: aspartate transaminase (AST) 756 U/L (NR 13 - 35 U/L), alanine aminotransferase (ALT) 611 U/L (NR 7 - 38 U/L), alkaline phosphatase (ALP) 192 IU/L (NR 32 - 117 U/L), total bilirubin 4.8 mg/dL (NR 0.2 - 1.3 mg/dL), and direct bilirubin 2 mg/dL (NR < 0.2 mg/dL). A viral hepatitis panel and secondary liver markers, including an autoimmune panel and serum ceruloplasmin level, returned normal. Ferritin was elevated at 2,346 ng/ml (NR 14.7 - 205.1 ng/ml) as was the transferrin saturation 44% (NR 15% - 57%), raising some concern for hemochromatosis.

**Figure 1:**
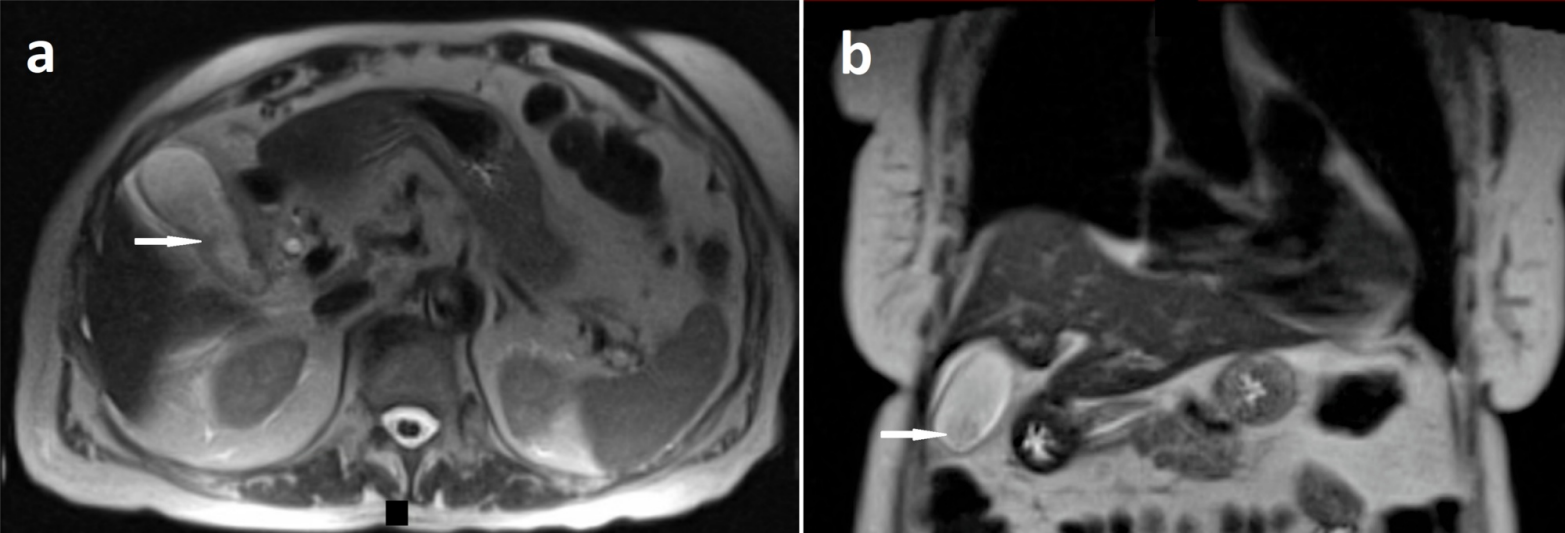
Magnetic resonance imaging (MRI) of the abdomen showing sludge in the gallbladder Axial (a) and coronal (b) sections of T2-weighted contrast-enhanced abdominal MRI showing layering sludge in the gallbladder (white arrows) with gall bladder wall thickening and trace pericholecystic fluid.

**Figure 2:**
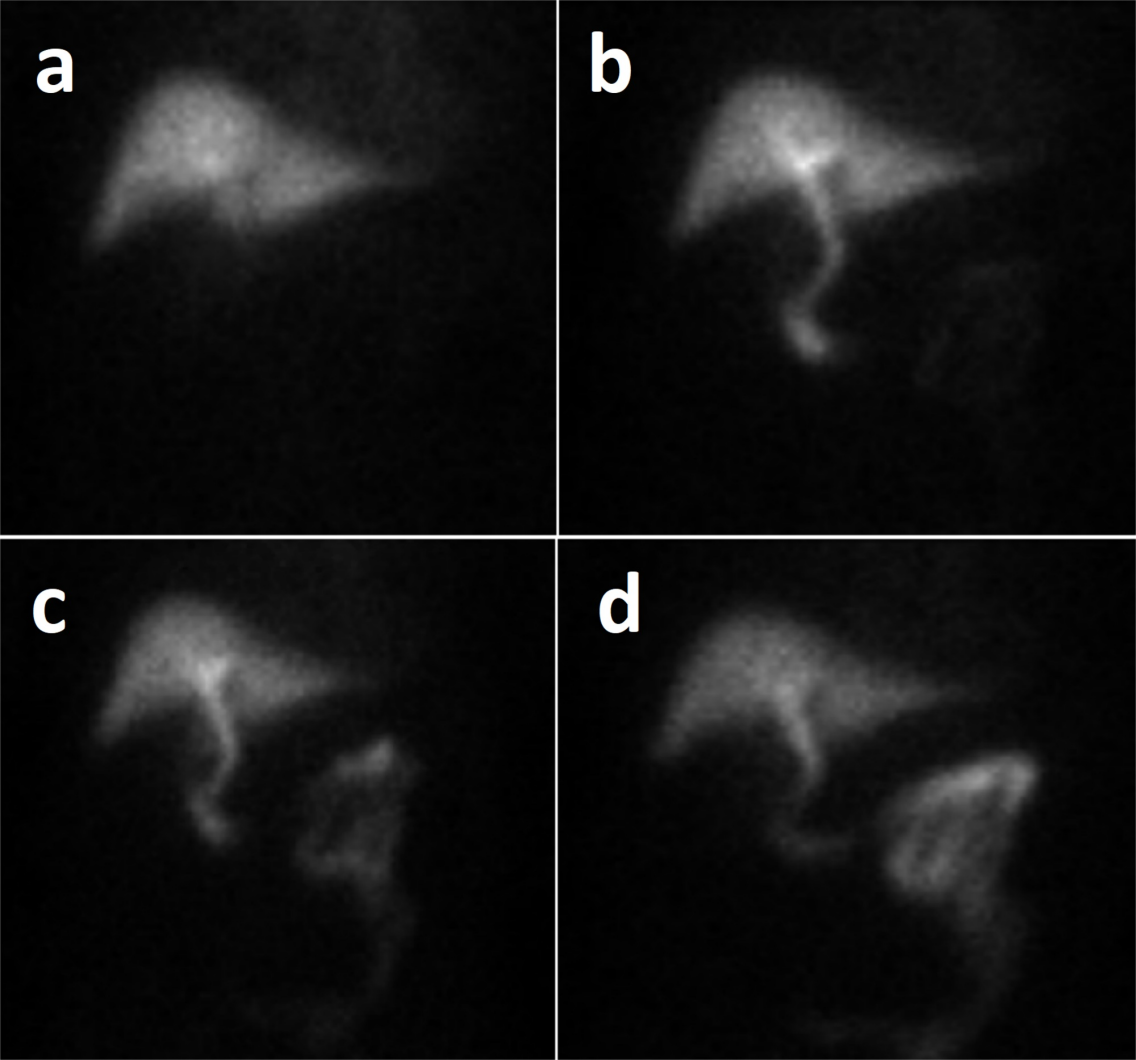
Dynamic hepatobiliary scintigraphy (HIDA) scan images of our patient taken at 5 minutes (a), 20 minutes (b), 40 minutes (c), and 60 minutes (d) intervals. The gall bladder could not be visualized, even after 60 minutes (d).

Computed tomography (CT) of the abdomen without contrast was non-revealing. This was followed by an ultrasound of the right upper quadrant which was remarkable for gallbladder wall thickening and sludge but was otherwise unremarkable with no intra- or extrahepatic biliary duct dilatation. Murphy’s sign was negative sonographically, and the common bile duct was reported to be 2.5 mm in diameter. Magnetic resonance cholangiopancreatography (MRCP) again demonstrated gallbladder wall thickening in addition to pericholecystic fluid and sludge in the gall bladder (Figure 1). Hepatobiliary iminodiacetic acid (HIDA) scan was consistent with acute cholecystitis as the gall bladder could not be visualized on the scan (Figure 2).

The surgery team was consulted, and given the HIDA scan findings, a laparoscopic cholecystectomy was scheduled. Intraoperatively, a 1 cm perforated duodenal ulcer sealed by the body of an inflamed and edematous gallbladder with no evidence of fistulation was identified. Cholecystectomy and a modified Graham patch omentopexy were performed without any complications. Biopsies later returned negative for malignancy. Helicobacter pylori immunoglobulin G (H. pylori IgG) antibody tested positive, and the patient was started on triple therapy (i.e., amoxicillin, clarithromycin, and pantoprazole). 

The postoperative course was uneventful. She started regaining her appetite and was able to tolerate a regular diet over the course of the next few days. Liver enzymes downtrended with a reported AST of 102 U/L, ALT of 120 U/L, ALP of 91 IU/L, and total bilirubin of 1.5 mg/dL on the day of discharge.

## Discussion

Acute cholecystitis is an inflammatory process probably mediated by the mucosal toxin, lysolecithin, a product of lecithin [10], as well as bile salts and platelets activating factor. An increase in prostaglandin synthesis amplifies the inflammatory process [11].

Peptic ulcer perforation can complicate 2% - 10% of patients with peptic ulcer disease [12] in the following anatomic locations: duodenum (60%), gastric antrum (20%), and gastric body (20%) [13]. The usual presentation of a perforated peptic ulcer is the sudden onset of acute abdominal pain and diffuse tenderness on physical examination. Other complications of peptic ulcer disease include bleeding and obstruction [14-15]. Acute acalculous cholecystitis is a rare complication of this disease and very few cases have been reported, especially in the setting of a perforated ulcer.

Acalculous cholecystitis is difficult to diagnose because findings of right upper quadrant pain, fever, leukocytosis, and abnormal liver function studies are non-specific and cannot differentiate it from the other types of cholecystitis. It is commonly encountered in critically and acutely ill patients. It is frequently overlooked due to the rarity of its occurrence. A delay in the diagnosis markedly increases the risk of clinical deterioration as it carries higher morbidity and mortality as compared to the other types [16]

Either ultrasonography or CT scan can be used for the diagnosis of acute acalculous cholecystitis; however, the former is preferred [17]. Although both modalities have similar sensitivities, the CT scan was found to have a higher specificity [18]. The combination of ultrasound and cholescintigraphy with cholecystokinin (HIDA scan) can also be used for confirmation. In the HIDA scan, technetium-labeled cholecystokinin is injected intravenously. Cholecystokinin is taken up by the hepatocytes and released into the bile, leading to the visualization of the intrahepatic/extrahepatic biliary ducts and gall bladder. Non-visualization of the gall bladder signifies a positive test result for cholecystitis.

The usual treatment of acute reactive acalculous cholecystitis is cholecystectomy and correction of the underlying pathology which, in this case, involved modified Graham patching of the duodenal perforation. However, in critically ill patients who cannot undergo surgery, cholecystostomy is an alternate option which can be done under local anesthesia and helps relieve distention of the gall bladder [19]. Cholecystectomy is typically done once the patient is stable enough to undergo the surgical procedure.

Alexakis et al. [8] evaluated the incidence of sealed perforated peptic ulcer disease among 5,539 patients who underwent laparoscopic cholecystectomy over a period of 10 years in a tertiary center in Greece. Only seven patients were found to have occult perforated peptic ulcer disease. Of them, four patients were treated with laparoscopic suturing and omental patch repair, two patients underwent pyloroplasty, and one patient required open omental patch repair.

Our case is unique in the sense that the patient presented with absolutely no symptoms of abdominal pain or tenderness. Additionally, the perforated duodenal ulcer and its contents were the likely culprits behind the inflammation of the gallbladder wall leading to acute cholecystitis. The lack of specificity of symptoms led to the delay in diagnosis in this patient but, fortunately, no adverse events were encountered.

## Conclusions

While acute cholecystitis secondary to cholelithiasis is a well-known entity, acute reactive acalculous cholecystitis is an uncommon occurrence, and as mentioned above, only a few cases have been reported. In addition to that, a perforated duodenal ulcer being the underlying cause of the gall bladder inflammation is even rarer. We are reporting this rare clinical presentation to increase awareness among clinicians regarding the possible clinical manifestations of the subtype and the different imaging modalities that can be helpful in reaching the diagnosis.
